# Identification and Molecular Characterization of Non-fermenting Gram-Negative Bacilli Exhibiting Carbapenem Resistance Isolated From Various Clinical Specimens in a Tertiary Care Hospital

**DOI:** 10.7759/cureus.107871

**Published:** 2026-04-28

**Authors:** Manish Kumar, Hitendra Singh, Nidhi Negi, Shekhar Pal, Shalabh Jauhari

**Affiliations:** 1 Microbiology, Government Doon Medical College, Dehradun, IND; 2 Microbiology, Veer Chandra Singh Garhwali Government Institute of Medical Science &amp; Research, Srinagar, IND

**Keywords:** acinetobacter baumannii, healthcare-associated infection, metallo-beta-lactamases (mbls), non‑fermenting gram‑negative bacilli (nfgnb), pseudomonas aeruginosa

## Abstract

Background: Non-fermenting Gram-negative bacilli (NFGNB), including *Pseudomonas aeruginosa *and *Acinetobacter baumannii*, are emerging nosocomial pathogens with intrinsic and acquired resistance to multiple antibiotics. Their increasing association with healthcare-associated infections (HAIs), especially in immunocompromised patients, poses a major therapeutic challenge, particularly due to carbapenem resistance mediated by metallo-β-lactamases (MBLs). This study aimed to isolate and identify NFGNB from various clinical specimens, assess their antimicrobial susceptibility patterns, detect carbapenem resistance phenotypically, and characterize MBL-producing isolates using molecular methods.

Methods: A descriptive and retrospective study was conducted in the Department of Microbiology, Government Doon Medical College, Dehradun, India, from September 2020 to February 2022. A total of 3,235 clinical specimens were processed for culture and identification. NFGNB were identified by colony morphology, Gram-staining, and biochemical tests. Antimicrobial susceptibility was performed using the Kirby-Bauer disc diffusion method. Screening for carbapenem resistance was done using imipenem and meropenem antibiotic discs, and MBL production was confirmed by the combined disc test (CDT) and modified Hodge test (MHT). Polymerase chain reaction (PCR) was used to detect *blaNDM-1*, *blaIMP*, and *blaVIM* genes in phenotypically positive isolates.

Results: Among 3,235 clinical samples, 1,924 (59.47%) samples showed culture positivity. A total of 220 (25.82%) isolates of NFGNB out of 852 (44.28%) isolates of Gram-negative bacilli were observed. The maximum proportion of NFGNB, excluding Gram-negative bacilli isolates, was reported from the pus (95, 89.62%) sample. *Pseudomonas* species (121, 55%) and *Acinetobacter* species (83, 37.22%) were the most common isolates. Imipenem resistance was seen in 96 (43.83%) isolates, and meropenem resistance in 76 (34.54%) isolates. Thirteen isolates (13.54%) were phenotypically MBL-positive, and PCR confirmed MBL genes in all 13 isolates. *blaNDM-1* was the most frequently detected gene. High resistance was observed in *Acinetobacter* isolates to carbapenems and fluoroquinolones, whereas minocycline showed the highest sensitivity.

Conclusion: The high prevalence of carbapenem-resistant and MBL-producing NFGNB underscores the urgent need for rapid detection, rational antibiotic use, and stringent infection control measures to limit the spread of multidrug-resistant organisms in healthcare settings.

## Introduction

*Pseudomonas aeruginosa* and other non-fermenting Gram-negative bacilli (NFGNB) are aerobic, non-sporing, opportunistic pathogens commonly found in soil, water, and occasionally as human gut commensals [1-4]. They do not ferment carbohydrates but derive energy through alternative metabolic pathways. Clinically significant NFGNB include *P. aeruginosa*, *Acinetobacter baumannii*, *Stenotrophomonas maltophilia*, and *Burkholderia cepacia*. In hospital environments, they are frequently isolated from ventilator equipment, patient linens, and the hands of healthcare personnel [5-7].

These organisms can cause a range of healthcare-associated infections (HAIs), including pneumonia, urinary tract infections, septicemia, meningitis, and surgical site infections [8]. They account for approximately 12-16% of all bacterial isolates in clinical microbiology laboratories. Risk factors for NFGNB infections include immunosuppression, indwelling catheters, invasive procedures, trauma, prolonged hospital stays, and broad-spectrum antibiotic use [1,5,9]. In patients with cystic fibrosis, organisms such as *P. aeruginosa*, *B. cepacia*, and *S. maltophilia* frequently cause chronic lung infections that are difficult to eradicate. NFGNB are known for their high intrinsic and acquired resistance to antibiotics, often due to genetic mutations or horizontal gene transfer [10].

*P. aeruginosa*, in particular, harbors a large resistance island comprising over 50 resistance genes. The mechanisms of antibiotic resistance in NFGNBs include the production of antibiotic-inactivating enzymes (e.g., extended-spectrum β-lactamases (ESBLs), AmpC, metallo-β-lactamases (MBLs)), efflux pumps, and mutations that alter antibiotic targets [11]. Carbapenems, often used as a last resort for multidrug-resistant Gram-negative infections, are increasingly compromised by carbapenemases - enzymes that hydrolyze carbapenems [12,13]. Among these, MBLs such as Verona integron-encoded (*VIM*) and imipenemase (*IMP*) types, encoded by *blaVIM* and *blaIMP* genes, are particularly concerning. These genes can be chromosomal or plasmid-mediated, facilitating rapid spread among Gram-negative organisms [14].

The presence of MBLs significantly limits therapeutic options, often necessitating the use of toxic or expensive alternatives with suboptimal outcomes. Early and accurate detection of carbapenem resistance and MBL production is therefore critical for guiding effective treatment and implementing infection control measures [14]. Due to a lack of regional molecular data on carbapenem-resistant NFGNB, the present study was undertaken at our tertiary care center to isolate and identify NFGNB from clinical specimens, evaluate their antimicrobial susceptibility patterns, detect carbapenemase production phenotypically, and identify MBL resistance genes using polymerase chain reaction (PCR).

## Materials and methods

Study design and setting

This was a descriptive and retrospective study conducted over a period of 18 months, from September 2020 to February 2022, in the Department of Microbiology at a tertiary care hospital in India, with the ethical approval (IEC no: GDMC/IEC/2021/03) from the Institutional Ethical Committee, Govt. Doon Medical College. The study focused on assessing the prevalence and antimicrobial resistance patterns of NFGNB, with particular emphasis on detecting metallo β-lactamase (MBL) production among carbapenem-resistant isolates.

Study population

This comprised all non-duplicate, clinically significant NFGNB isolated from various clinical specimens, including urine, pus, blood, sputum, cerebrospinal fluid (CSF), pleural fluid, ascitic fluid, peritoneal fluid, and throat swabs received from both inpatients (hospitalized for more than 48 hours) and outpatients. Patients with co-morbid conditions, such as burns or cystic fibrosis, which increase susceptibility to infections with *Pseudomonas* and other non-fermenters, were included. Exclusion criteria involved all non-lactose-fermenting members of the Enterobacteriaceae family, samples from patients hospitalized for less than 48 hours, and any subsequent carbapenem-resistant non-fermenting isolate from the same patient, whether from the same or different anatomical sites. All clinical specimens were processed using standard microbiological procedures in the microbiology laboratory of the tertiary care centre.

Sample size

Among the non-lactose fermenting organisms, the *P. aeruginosa* is one of the most common causative agents for nosocomial infections. The sample size is calculated based on the prevalence of MBL in *P. aeruginosa* in India, which ranges from 7% to 65% [5]. The formula for the calculation of the sample size is 4pq/d^2^, where p (prevalence) = 60, q = 100-p = (100-60=40), and d = standard error of difference, which ranges from 5% to 20% = 10%. Hence, the sample size is (4x60x40)/10^2^ = 96 imipenem-resistant isolates.

Data collection

Processing of Clinical Samples

Clinical samples were processed using standard microbiological techniques. For urine samples, clean-catch midstream specimens were collected and processed within two hours. Wet mount microscopy was done to detect pus cells, red blood cells, and microorganisms. For culture, the sample was streaked onto cystine-lactose-electrolyte-deficient (CLED) agar using a 4 mm loop and incubated at 37°C for 18-24 hours. Pus and wound swab samples were collected using sterile syringes or swabs and directly inoculated on blood agar, MacConkey agar, and brain-heart infusion (BHI) broth. After incubation at 37°C for 18-24 hours, the presence of pale colonies or turbidity in BHI prompted subculture. Direct smears were Gram-stained to visualize pus cells and organisms. Respiratory tract specimens, including sputum, bronchoalveolar lavage (BAL), and gastric aspirates, were collected in sterile containers. Sputum samples were assessed microscopically, and only those with >25 pus cells and <10 epithelial cells per high-power field were cultured. Samples were inoculated on blood, MacConkey, and chocolate agar and incubated at 37°C for 18-24 hours. Endotracheal tube tips were processed by rolling on culture plates. BAL and gastric aspirates were similarly processed with Gram staining and culture. Blood cultures were obtained from two separate venipuncture sites and inoculated into BacT/ALERT 3D-automated blood culture bottles. Once flagged positive, a drop of the sample was used for Gram-stained smears and subcultured onto blood and MacConkey agar. Sterile body fluids, such as CSF, were collected aseptically and transported within two hours. Smears were prepared in triple layers, Gram-stained, and cultured onto blood, MacConkey, and chocolate agar, with a portion also inoculated in BHI broth. Plates were incubated at 37°C for 18-24 hours. If no growth occurred on solid media but BHI showed turbidity, subculture was performed; the absence of growth on subculture indicated a sterile sample.

Identification

Identification of organisms was initially based on colony morphology on solid culture media. Pale-colored, non-lactose-fermenting colonies were suggestive of non-fermenters. *Pseudomonas* species appeared as large, flat, boat-shaped, non-lactose fermenting colonies on MacConkey agar and large, flat, beta-hemolytic colonies on blood agar. *Acinetobacter* species showed medium-sized, smooth, convex colonies with a slightly pink hue on MacConkey agar and exhibited a coccoid or coccobacillary appearance on Gram staining. *S. maltophilia* colonies were smooth, round, green with a darker olive-green center and a lighter green periphery. *Burkholderia* species presented either as non-mucoid white, opaque colonies with umbilicate or umbonate surfaces or as mucoid, white/yellow, shiny colonies with smooth surfaces. Final identification was done using a panel of biochemical tests, including catalase, oxidase, pigment production on nutrient agar, mannitol motility agar, growth at 42°C, decarboxylase test, Hugh-Leifson's oxidative-fermentative test, nitrate reduction, indole production, citrate utilization, urease test, triple sugar iron (TSI) agar, arginine dihydrolase, and O/F utilization of sugars, such as mannitol, lactose, and dextrose.

**Figure 1:**
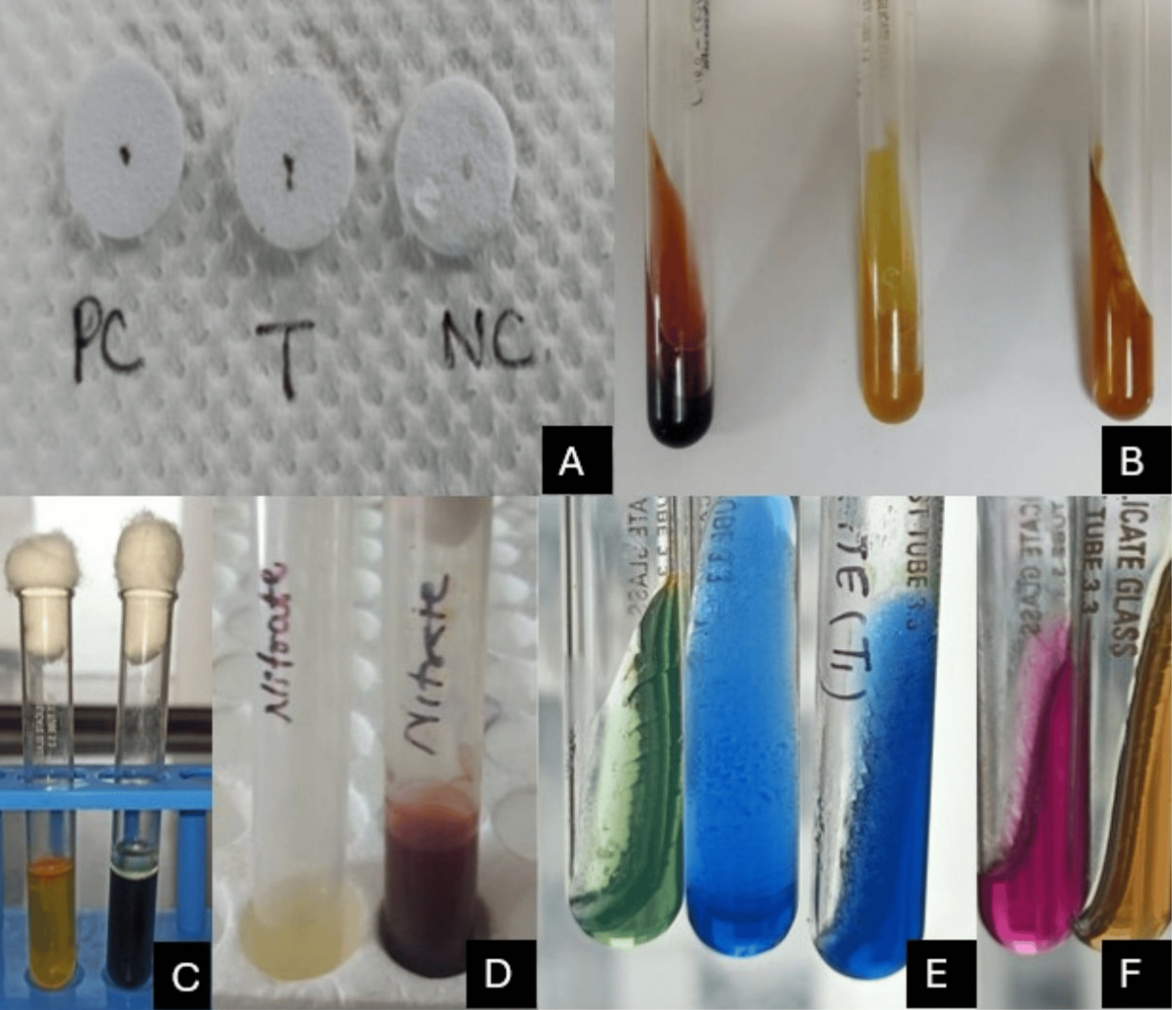
Biochemical characterization of NFGNB using a series of diagnostic tests. A: Oxidase test (PC - positive control, T - test organism, NC - negative control); B: Triple sugar iron agar test; C: Hugh Leifson's oxidative & fermentative test; D: Nitrate reduction test; E: Citrate utilization test; F: Urea hydrolysis test NFGNB: non-fermenting Gram-negative bacilli

Figure 1 demonstrates the biochemical characterization of NFGNB using a series of diagnostic tests. The oxidase test (A) shows a positive reaction in the test and positive control discs, indicative of oxidase-producing organisms, such as *Pseudomonas *species. The TSI agar (B) displays an alkaline slant and butt or no change, suggesting the absence of carbohydrate fermentation typical of non-fermenters. Hugh Leifson's OF test (C) shows oxidative metabolism with acid production only in the open tube. The nitrate reduction test (D) shows a red color, confirming the ability to reduce nitrate - a feature seen in both *Pseudomonas *and *Acinetobacter *species. The citrate utilization test (E) is positive with a blue color change, indicating the organism's ability to utilize citrate as the sole carbon source. Finally, the urea hydrolysis test (F) reveals urease activity in some isolates (pink color), which is commonly associated with *Acinetobacter *spp. These test results collectively support the identification and differentiation of key non-fermenters in clinical microbiology.

Antibiotic Susceptibility Testing

Antibiotic susceptibility testing was performed using the Kirby-Bauer disc diffusion technique according to the Clinical & Laboratory Standards Institute (CLSI) 2022 guidelines. The following antibiotics were used for susceptibility testing: ceftazidime (30 µg), ciprofloxacin (5 µg), levofloxacin (5 µg), gentamicin (10 µg), amikacin (30 µg), piperacillin-tazobactam (100/10 µg), meropenem (10 µg), imipenem (10 µg), minocycline (30 µg), tobramycin (10 µg), and trimethoprim-sulfamethoxazole (1.25/23.75 µg). The *P. aeruginosa* ATCC 27853 strain and *Acinetobacter baumannii* ATCC 19606 strain were used as the quality control. All the discs were procured commercially from Hi-media Laboratories Limited (Maharashtra, India). The diameter of the zone of inhibition was measured and interpreted according to the CLSI 2022 guidelines [15].

Detection of MBL-Producing Organisms

Screening for MBL production was done using imipenem (10 µg) and meropenem (10 µg) antibiotic discs by the Kirby-Bauer disk diffusion method. A zone diameter of < 16 mm indicated resistance, while > 22 mm for imipenem and > 18 mm for meropenem were considered sensitive. Phenotypic confirmation was done using the combined disc test (CDT) and modified Hodge test (MHT). In CDT, imipenem-resistant isolates were spread on Mueller-Hinton agar, and both imipenem and imipenem+EDTA discs were placed 4-5 cm apart. An increase of ≥ 7 mm in the zone diameter with IPM+EDTA compared to IPM alone indicated MBL production. The MHT involved streaking *Escherichia coli* ATCC 25922 on Mueller-Hinton agar (MHA), placing an imipenem disc centrally, and streaking the test strain from the disc to the plate edges. A cloverleaf-shaped zone indicated carbapenemase activity.

Molecular Detection

DNA extraction was performed using the QIAGEN kit (QIAGEN N.V., Hilden, Germany). Bacterial colonies were suspended in buffer ATL and proteinase K, followed by sequential addition of buffer AL and ethanol. The lysate was loaded onto a QIAamp spin column, washed with buffer AW1 and AW2, and the DNA was eluted with buffer AE. The purified DNA was stored at 4-8°C. PCR was used to detect carbapenemase genes (NDM-1, IMP, and VIM) using specific primers (Sigma-Aldrich, St. Louis, MO). For the molecular detection of carbapenemase genes, specific primers were used targeting *NDM-1*, *VIM*, and *IMP* genes. The forward and reverse primer sequences for *NDM-1* were 5’-GTAGTGCTCAGTGTCGGCA-3’ and 5’-GGGCAGTCGCTTCCAACGGT-3’, respectively, yielding a 475 bp product. The *VIM *gene was amplified using degenerate primers with sequences 5’-SGRTRSRTGGRCRCATASCRCS-3’ (forward) and 5’-TCSSCRGRACCRSAGCRCACR-3’ (reverse), generating a 360 bp product. For *IMP*, the primers were 5’-GRAASAGARTRGCSTAST-3’ (forward) and 5’-CSACRTTSTCTRRAGTGS-3’ (reverse), yielding a 181 bp product. Each primer was synthesized at a 0.025 µmol scale. PCR amplification was carried out using AmpliTaq Gold™ 360 Master Mix (Thermo Fisher Scientific, Waltham, MA). The 25 µL reaction mixture consisted of 12.5 µL of 10x PCR buffer, 8.0 µL of molecular-grade water, 0.5 µL of MgCl₂ enhancer, 0.5 µL each of forward and reverse primers, and 3 µL of template DNA. The PCR program included an initial denaturation at 94°C for five minutes, followed by 30 cycles of denaturation at 94°C for one minute, annealing at 54°C for one minute, and extension at 72°C for 1.5 minutes. The final PCR cycle was completed in 1 hour and 38 minutes. Post-amplification, 20 μL of each PCR product was analyzed using 2% agarose E-gel (Thermo Fisher Scientific), which contained cyan orange dye (0.2 mg/mL). Electrophoresis was performed using a DNA ladder ranging from 50 to 2,500 base pairs, prepared by mixing 10 μL of ladder with 10 μL of buffer. For each sample, the gel loading mixture consisted of 10 μL amplified DNA, 3.3 μL loading dye, and 6.7 μL buffer, totaling 20 μL. Each sample was run in triplicate for *NDM-1*, *VIM*, and *IMP *primers. The 11-well E-gel cassette had the molecular weight marker (ladder) in the M well and test samples in wells 1-10. The run was conducted for 12 minutes using the E-gel Power Snap Electrophoresis Device (Thermo Fisher Scientific), after which gel images were captured using the E-gel Power Snap Camera and saved to a storage device.

**Figure 2:**
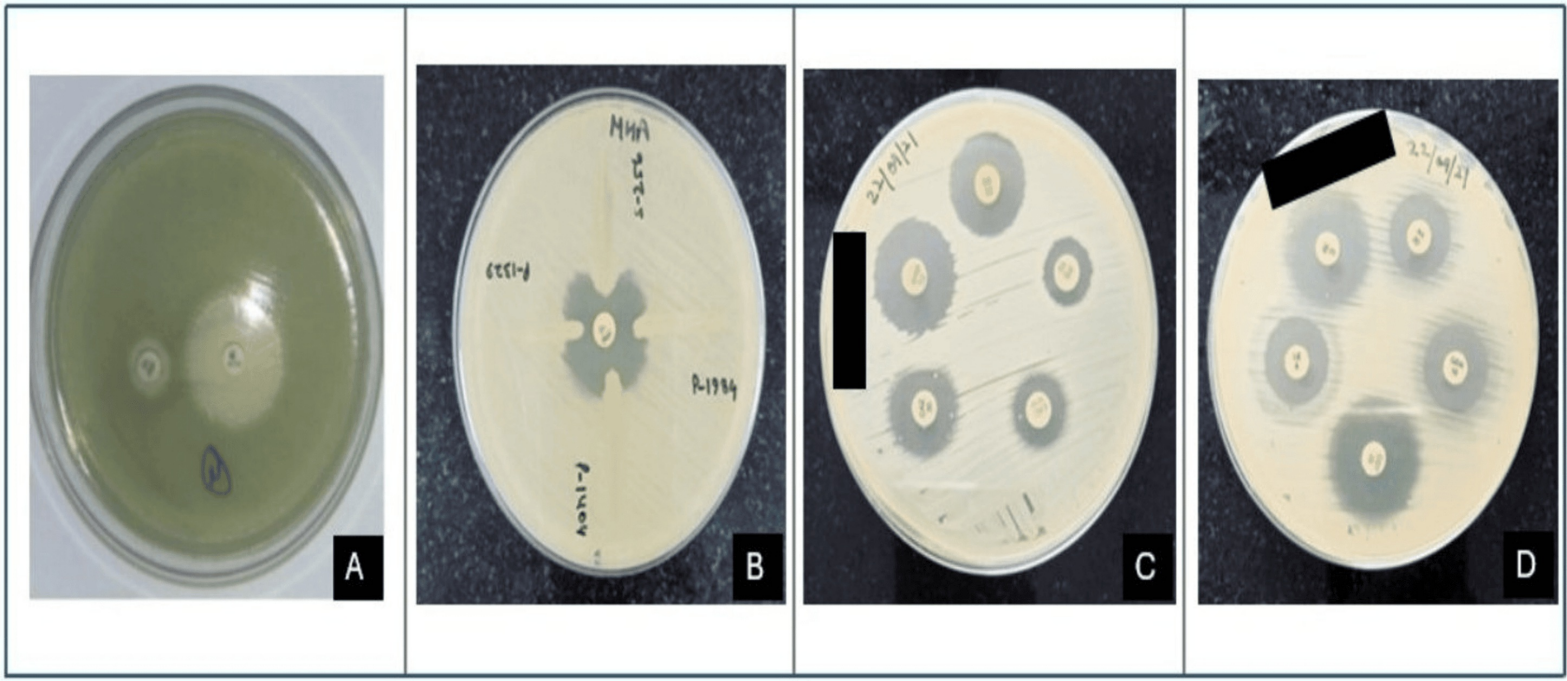
Phenotypic methods used for the detection of carbapenemase production and antimicrobial susceptibility testing in NFGNB. A: Combined disc test (IPM and IPM+EDTA) (IPM - Imipenem, EDTA - Ethylenediaminetetraacetic acid); B: Modified Hodge test; C, D: Antibiotic disc diffusion testing NFGNB: non-fermenting Gram-negative bacilli

Figure 2 depicts phenotypic methods used for the detection of carbapenemase production and antimicrobial susceptibility testing in NFGNB. In image A, the CDT shows a ≥ 7 mm increase in the inhibition zone around the imipenem-EDTA disc compared to imipenem alone, indicating a positive result for MBL production. Image B illustrates the MHT, where the cloverleaf-shaped indentation toward the central imipenem disc signifies carbapenemase enzyme production. Images C and D display standard antibiotic disc diffusion testing (Kirby-Bauer method) showing varying zones of inhibition, which reflect the susceptibility profiles of the isolates against different antibiotics. These tests are crucial for detecting drug-resistant pathogens and guiding appropriate antimicrobial therapy.

Data analysis

Descriptive statistical analysis was performed using Statistical Product and Service Solutions (SPSS, version 22; IBM SPSS Statistics for Windows, Armonk, NY). Data were analyzed based on the presence of resistance detected by phenotypic methods, including the number and percentage of isolates showing MBL production. Similarly, the distribution of carbapenemase-encoding genes *(blaNDM-1*, *blaVIM*, and *blaIMP*) detected by PCR was expressed in terms of frequency and percentage. To assess the statistical significance of associations between phenotypic resistance and gene presence, chi-square analysis was applied, and p-values were calculated. A p-value of < 0.05 was considered statistically significant.

## Results

**Figure 3:**
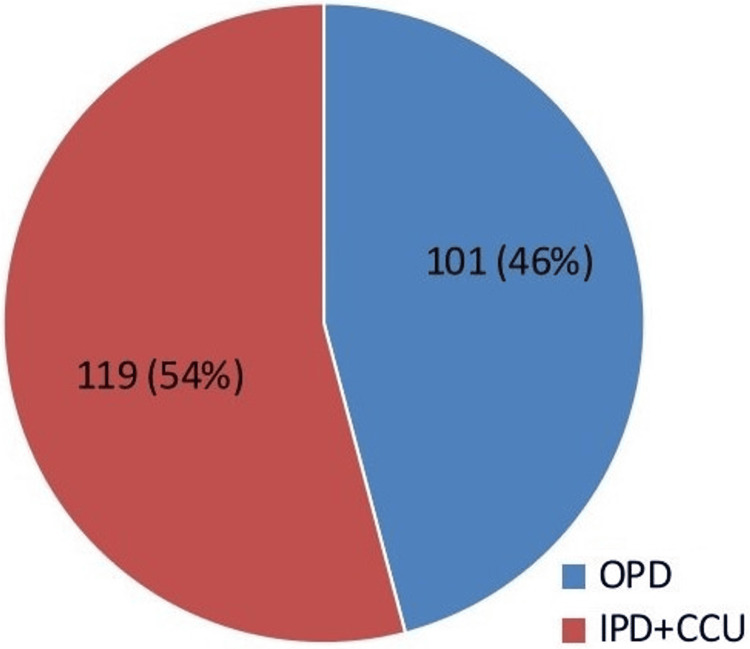
Distribution of patients (N = 220) OPD/IPD/CCU-wise. OPD: outpatient department, IPD: inpatient department, CCU: critical care unit

Among 3,235 clinical samples, 1,924 (59.47%) samples showed culture positivity. A total of 220 (25.82%) isolates of NFGNB out of 852 (44.28%) isolates of Gram-negative bacilli were observed. The maximum proportion of NFGNB, excluding Gram-negative bacilli isolates, was reported from pus (95, 89.62%) samples, followed by sputum (75.67%) and body fluids (71.42%). The most commonly isolated NFGNB was *Pseudomonas* species, predominating in pus (66.31%) and urine (59.42%), while *Acinetobacter* species was more frequently isolated from body fluids (83.33%) and blood (76.92%). Less frequently detected species included *Burkholderia*, *Stenotrophomonas*, and *Achromobacter*. These findings highlight the predominance of *Pseudomonas* and *Acinetobacter* species in non-fermenter infections, particularly in wound and respiratory samples (Table 1). Additionally, 54% of patients were admitted (IPD+CCU), while 46% were managed on an outpatient basis (OPD) (Figure 3).

**Table 1:** Distribution and species-wise frequency of non-fermenter Gram-negative bacilli (NFGNB) isolated from various clinical specimens. Total number of specimens: 3,235; total number of NFGNB isolates: 220; HVS: high vaginal swab sample; GNB: Gram-negative bacilli

Specimens	Total growth	GNB	NFGNB excluding Enterobacteriaceae	% of NFGNB excluding Enterobacteriaceae out of GNB	NFGNB				
					*Pseudomonas *species	*Acinetobacter *species	*Burkholderia *species	*Stenotrophomonas *species	*Achromobacter *species
Urine (2307)	1272	625	69	11.04%	41 (59.42%)	24 (34.78%)	01 (1.44%)	02 (2.89%)	01 (1.44%)
Pus (386)	330	106	95	89.62%	63 (66.31%)	28 (29.47%)	01 (1.05%)	02 (2.10 %)	01 (1.05%)
Sputum (85)	80	37	28	75.67%	15 (53.57%)	10 (35.71%)	0	03 (10.71%)	0
Blood (274)	162	52	13	25.0%	0	10 (76.92%)	01 (7.69%)	01 (7.69%)	01 (7.69%)
Body fluids (71)	30	07	06	71.42%	01 (16.66%)	05 (83.33%)	0	0	0
HVS (112)	50	25	09	36.0%	01 (11.11%)	06 (66.66%)	01 (11.11%)	01 (11.11%)	0

Table 2 summarizes the results of carbapenem resistance screening using imipenem and meropenem discs via the Kirby-Bauer disc diffusion method. Resistance to imipenem was observed in 43.83% of isolates, with a statistically significant association (p = 0.05), suggesting a high prevalence of carbapenem resistance. Meropenem resistance was seen in 34.54% of isolates, though no chi-square value was provided. These findings underscore the need for further confirmatory testing for carbapenemase production among resistant isolates.

**Table 2:** Screening tests for carbapenemase enzyme production in a total of 220 NFGNB-positive isolates with both imipenem and meropenem antibiotic discs. NFGNB: non-fermenting Gram-negative bacilli

Antibiotic disc	Resistance	Sensitive	Chi-square (χ²)	p-value
Imipenem (10 µg)	96 (43.83%)	124 (56.36%)	3.81	0.05
Meropenem (10 µg)	76 (34.54%)	144 (65.45%)		

Both the CDT and MHT detected carbapenemase production in 13 isolates (13.54%). The concordance between both tests suggests their reliability in identifying metallo-β-lactamase producers among NFGNB. These results support the importance of confirmatory testing in guiding antimicrobial stewardship (Table 3).

**Table 3:** Combined disc test and modified Hodge test for carbapenemase enzyme production for all 96 isolates found to be resistant to the imipenem disc by screening test.

Test	No. of Isolates Tested	Isolates Positive
Combined disc test (IMP+EDTA)	96 (43.83%)	13 (13.54%)
Modified Hodge test	96 (43.83%)	13 (13.54%)

Table 4 compares the distribution of carbapenemase-producing and non-producing NFGNBs between *Pseudomonas* and *Acinetobacter* species. A statistically significant difference (p = 0.01) was observed, with *Acinetobacter* showing a higher frequency of carbapenemase production. Among gene-positive isolates, *blaNDM-1* was the most commonly detected gene, followed by *blaVIM* and *blaIMP*. However, the distribution of resistance genes between the two species was not statistically significant (p = 0.476), suggesting similar genetic profiles for carbapenem resistance among these pathogens.

**Table 4:** Distribution of carbapenemase-producing NFGNB and associated resistance genes. NFGNB: non-fermenting Gram-negative bacilli

Variables		Pseudomonas species	Acinetobacter species	Chi-square value (χ²)	p-value
Non-fermenter species	Non-carbapenemase producing NFGNB	121	83	6.55	0.01
	Carbapenemase-producing NFGNB	03	10		
Total carbapenemase-producing NFGNB	bla_NDM-1 _alone	02	06	0.127	0.476
	bla_VIM _alone	0	01		
	bla_NDM-1 _+ bla_IMP_	01	0		

**Figure 4:**
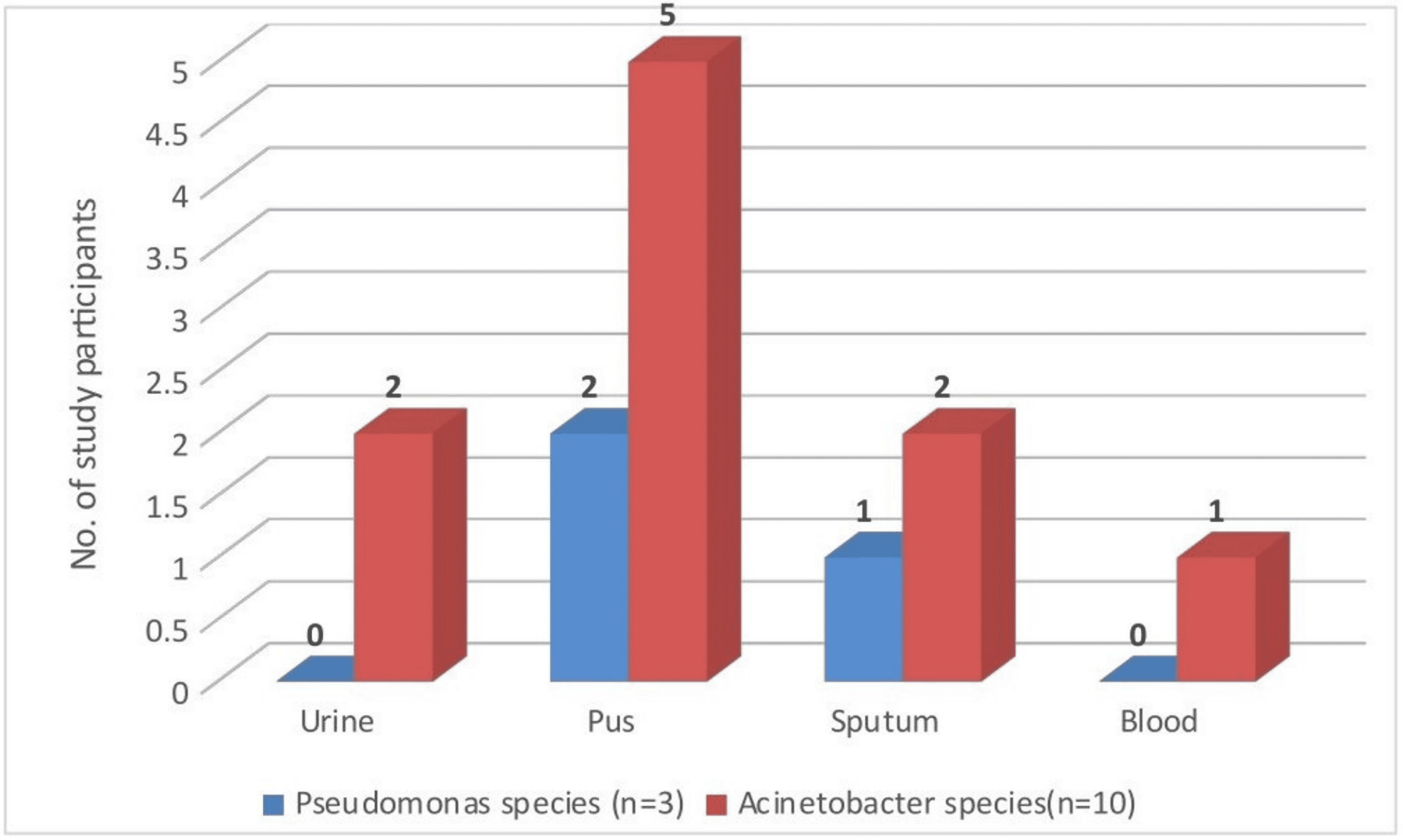
Carbapenemase production among clinical isolates, specimen-wise. Urine: 02 isolates of *Acinetobacter *species; pus: 02 isolates of *Pseudomonas *species and 05 of *Acinetobacter *species; sputum: 01 isolate of *Pseudomonas *species and 02 of *Acinetobacter *species; blood: 01 isolate of *Acinetobacter *species

Among the 13 MBL-positive isolates, *Pseudomonas* species (n = 3) were primarily isolated from pus (2) and sputum (1), while *Acinetobacter* species (n = 10) were most frequently isolated from pus (5), followed by urine (2), sputum (2), and blood (1) (Figure 4).

The antibiotic resistance patterns among NFGNB were also observed. *Pseudomonas* species showed notable resistance to imipenem (32.23%) and levofloxacin (29.75%), while *Acinetobacter* species exhibited high resistance to both imipenem (59.03%) and meropenem (45.78%). *Burkholderia *spp. demonstrated considerable resistance across multiple agents (50-75%), particularly to gentamicin, amikacin, and cotrimoxazole. *Stenotrophomonas* species showed the highest resistance to imipenem (66.66%) and varying degrees of resistance to other antibiotics. These findings reflect a high prevalence of multidrug resistance, especially among *Acinetobacter* isolates, emphasizing the need for targeted antimicrobial stewardship (Table 5).

**Table 5:** Overall antibiotic resistant pattern among non-fermenters. 121 *Pseudomonas *species; 83 *Acinetobacter *species; 04 *Burkholderia *species; 09 *Stenotrophomonas *species R: Resistance

Antimicrobial agents	*Pseudomonas* species (121, 55%)	*Acinetobacter* species (83, 37.72%)	*Burkholderia* species (04, 1.81%)	*Stenotrophomonas *species (09, 4.09%)
	R (%)	R (%)	R (%)	R (%)
Ceftazidime	28 (23.14%)	33 (39.75%)	02 (50%)	-
Gentamicin	27 (22.31%)	26 (31.32%)	03 (75%)	-
Piperacillin-tazobactum	17 (14.04%)	30 (36.14%)	02 (50%)	-
Amikacin	23 (19%)	20 (24.09%)	03 (75%)	-
Ciprofloxacin	26 (21.48%)	15 (18.07%)	02 (50%)	-
Imipenem	39 (32.23%)	49 (59.03%)	02 (50%)	06 (66.66%)
Levofloxacin	36 (29.75%)	18 (21.68%)	02 (50%)	02 (22.22%)
Minocycline	-	0	01 (25%)	03 (33.33%)
Meropenem	32 (26.44%)	38 (45.78%)	03 (75%)	03 (33.33%)
Tobramycin	11 (9.09%)	03 (3.61%)	01 (25%)	03 (33.33%)
Cotrimoxazole	-	26 (31.32%)	03 (75%)	03 (33.33%)

## Discussion

NFGNB, once regarded as less significant, have now emerged as major nosocomial pathogens responsible for hospital-acquired and opportunistic infections. The indiscriminate use of antibiotics has led to a surge in multidrug-resistant strains, resulting in prolonged hospital stays, increased financial burden, and rising morbidity and mortality.

In this study, NFGNB were identified in 220 (25.82%) clinical specimens, consistent with findings by Sharma et al. [16] and Yadav et al. [17], who reported incidences of 25.6% and 27.1%, respectively. The majority (89.62%) of NFGNB isolates were recovered from pus samples, aligning with the study by Hemwani et al., who reported 96.3% *Pseudomonas *isolates in pus [18]. However, this prevalence is higher than that reported by Malini et al. [19] and Gokale et al. [20], who documented rates of 62.2% and 58.4%, respectively, suggesting that NFGNB are key agents in localized pyogenic infections.

*Pseudomonas* species was the predominant isolate (55%) in our study, correlating with Kaur et al. [21]. Its high prevalence in critical care settings may be attributed to colonization in ICU environments, instruments, and healthcare personnel. Other studies by Yadav et al. [17] and Paul et al. [22] reported lower prevalences of 40% and 29.67%, respectively. *Acinetobacter* species was the second most common NFGNB (37.22%), similar to findings by Manjunath et al. [23] and Kaur et al. [21], who reported 36.3% and 35%, respectively. In contrast, Yadav et al. reported *A. baumannii* (44%) as the most frequent NFGNB, likely due to inclusion of hospitalized multidrug-resistant-suspected cases [17]. Paul et al. also found a higher incidence (54.4%) of *Acinetobacter* among severely ill and immunocompromised patients [22]. This distribution can be attributed to the natural adaptability of *Pseudomonas* in moist hospital environments, such as sinks, catheters, ventilators, and nebulizers, and the ability of *Acinetobacter* to persist on dry surfaces and develop resistance rapidly.

Our study found that 54.09% of NFGNB isolates were from hospitalized patients, including those from obstetrics, medicine, surgery, and ICUs, consistent with Kaur et al. [21], who found 56.9% from IPD and critical care units. Juyal et al. and Jayapriya et al. also reported high ICU isolations (67% and 58%, respectively), confirming the hospital-associated nature of these organisms [24,25].

The emergence of MBLs presents a major challenge as they confer resistance to carbapenems and are often plasmid-mediated, enabling rapid spread. Among 220 NFGNB isolates, 96 (43.83%) were resistant to imipenem and 76 (34.54%) to meropenem. The chi-square value was 3.81, and the p-value was 0.05, showing statistical significance. These results are similar to those of Manjunath et al. [23], who reported 44.3% imipenem resistance, but differ from Kaur et al. [21], where 71.4% of isolates were sensitive to imipenem.

Phenotypic MBL detection using CDT and MHT showed that 13 (13.54%) of the 96 imipenem-resistant isolates were positive in both tests. This is consistent with findings by John et al. [26], who reported 14.8% positivity. However, Jayalakshmi et al. [27] and Jesudasan et al. [28] reported higher rates of 33% and 56%, respectively. In our study, both phenotypic methods showed equal sensitivity. Among MBL producers, *Acinetobacter* species accounted for 12.04% and *Pseudomonas* species for 2.47%, closely resembling the study by Namaei et al. [29], which reported 13.3% positivity in *Acinetobacter*. The chi-square value was 6.55 with a p-value of 0.01, indicating strong statistical significance. The higher prevalence of MBL genes in *Acinetobacter* may be explained by its genomic plasticity, ability to acquire resistance genes via horizontal gene transfer, and the presence of insertion sequences that facilitate mobilization of resistance elements.

Carbapenemase-producing *Acinetobacter *isolates were mostly from pus, followed by urine, sputum, and blood, while *Pseudomonas* producers were recovered from pus and sputum. These findings reinforce the clinical importance of NFGNB in hospital-acquired infections and the urgency for routine surveillance. Antimicrobial resistance patterns varied. *Pseudomonas* species exhibited high resistance to imipenem (32.23%) and levofloxacin (29.75%). *Acinetobacter* species showed 59.03% resistance to imipenem and 45.78% to meropenem, with minocycline being relatively effective. These findings are consistent with Juyal et al. [24], who reported 27.05% and 31.13% resistance in *Pseudomonas* and *Acinetobacter*, respectively, though in contrast with Tripathi et al. [2], who found ceftazidime had the highest resistance (36%). Our results differ from those of Mohammad Rahbar [5] and Malini et al., who observed imipenem sensitivity as high as 78.9% and 94.2%, respectively [19]. Tobramycin and piperacillin-tazobactam were the most effective drugs against *Pseudomonas*, differing from Juyal et al. [24], where amikacin (72.3%) was most effective.

*Burkholderia *species showed high resistance to meropenem (75%), aminoglycosides, and cotrimoxazole, which contradicts Yadav et al. [17], who reported 66.7% susceptibility to meropenem and cotrimoxazole. *S. maltophilia* demonstrated 66.66% resistance to imipenem and 77.77% sensitivity to levofloxacin, aligning with Namaei et al. [29], but not with Yadav et al. [17], who found only 25% sensitivity.

Genotypic detection of *blaNDM*, *blaVIM*, and *blaIMP *genes via PCR confirmed MBL gene presence in all 13 phenotypically positive isolates. Similar discrepancies between phenotypic and molecular findings were reported by Yousefi [11] and Pena et al. [13], possibly due to other resistance mechanisms or undetected MBL variants. Behera et al. also noted that the combined disc test is as sensitive as the E-test but more economical [30].

Among the three *Pseudomonas* isolates, two were *blaNDM*-positive and one co-harbored *blaIMP *and *blaNDM-1*. This differs from Fallah et al. [4], who found six isolates positive for *blaIMP *but none for *blaVIM*. Aghamiri et al. reported 9% for *blaIMP *and 33% for *blaVIM* [6], while Hirataka et al. linked *blaIMP *with more severe infections [7]. Among 10 *Acinetobacter* isolates, one was *blaVIM-*positive, and six were *blaNDM-1*-positive. Uma Karthika et al. found 42% *blaIMP *positivity and no *blaVIM* in *A. baumannii* [10].

These findings highlight the increasing threat posed by multidrug-resistant NFGNB, particularly in nosocomial settings where immunocompromised patients, invasive procedures, prolonged hospital stays, and antibiotic pressure create ideal conditions for the selection and spread of resistant strains. The study underscores the urgent need for implementing hospital-specific antibiotic stewardship programs, rapid diagnostics, and stringent infection control practices to mitigate the spread of these pathogens. The rise of MBLs highlights a growing threat of pan-resistance. Therefore, strict antibiotic stewardship and infection control are essential to limit the spread of these highly resistant nosocomial pathogens.

Although the study highlights the core concept of antibiotic stewardship and to follow strict IPC protocols, it also had some limitations. Firstly, a multicentric study could have provided a better understanding of the resistance pattern. Secondly, only 13 isolates that produced the carbapenemase enzyme had their MBL genes tested. The precision of the results would have increased with more isolates or a longer study period. Thirdly, because of time constraints and financial constraints, we could not proceed with target sequencing. It could have given us better insight about research topic. Finally, the presence of carbapenemase activity in bacterial isolates from animals as well could have given us a more accurate picture of the research.

## Conclusions

To conclude, out of 3,235 clinical samples, 220 NFGNB were isolated, with the majority recovered from pus samples. *Pseudomonas* and *Acinetobacter* species were the predominant pathogens, frequently associated with hospital-acquired infections and multiple risk factors, particularly urinary catheterization. Notably, high resistance was observed to carbapenems and fluoroquinolones, with *Acinetobacter* showing 59.03% resistance to imipenem. However, minocycline and tobramycin remained effective against most isolates, with minocycline demonstrating 100% sensitivity.

Furthermore, 13 imipenem-resistant isolates were confirmed as MBL producers by phenotypic tests and subjected to PCR. Genotypic analysis revealed the presence of *blaNDM-1*, *blaIMP*, and *blaVIM *genes, with* blaNDM-1* being the most common. The emergence of these carbapenemase genes underscores the urgent need for routine surveillance, judicious antibiotic use, and stringent infection control practices to combat the spread of multidrug-resistant NFGNB in healthcare settings.
